# Analysis of internet use behaviors among clinical medical students in China

**DOI:** 10.1186/1472-6920-14-67

**Published:** 2014-04-02

**Authors:** Hua Yang, Yunxiang Chen, Liqiang Zheng, Xin Xu, Xia Cao

**Affiliations:** 1 Library of Shengjing Hospital, China Medical University, No. 36 Sanhao Street, Heping District, Shenyang, Liaoning Province 110004, China

## Abstract

**Background:**

The availability of internet-based information resources is increasing and the appropriate use of such resources is an important subject for clinical medical students. The aims of this study were to investigate the behaviors of clinical medical students regarding the use of internet-based activities, to analyze the behavior and characteristics of the students’ information demands, and to discuss the behaviors and time preferences related to internet use of students with different levels of education.

**Methods:**

Librarians obtained real-time feedback from 999 clinical medical students to record online activities. The data was recorded in a standard form and then analyzed statistically.

**Results:**

There were significant differences in the use of the internet for learning activities among the different groups of clinical medical students (P < 0.0001). Learning accounted for 73.5% of all internet use for doctoral candidates, 47.6% of internet use for master’s candidates, 28.7% of internet use for seven-year undergraduate students, and 14.1% of use for five-year undergraduate students. There was also a significant difference in the proportions of leisure and e-commerce activities among the student groups (P < 0.0001), with five-year students displaying the highest total proportion of these activities (59.4% and 18.8%). Internet use for entertainment activities was the same for all groups of clinical medical students. Time of day of internet use was consistent across all student groups, but internet use differed by day of the week (P < 0.01). There was no difference among the time of day of internet use for learning, leisure and entertainment activities during a single day (P > 0.05), but e-commerce activities varied according to time of day (P < 0.05). Learning and e-commerce activities by clinical medical students did not vary by day of the week (P > 0.05), but the distributions of leisure and entertainment activities were different according to day of the week (P < 0.05).

**Conclusions:**

A stronger demand for learning is associated with a higher academic level of clinical medical students. Differences exist among student groups regarding internet use behaviors and internet use during different time periods.

## Background

The availability of internet-based information resources has increased rapidly and clinical medical students frequently use the internet to obtain information. Consequently, the internet has become an important component of quality-oriented education in medical colleges, as it encourages students to obtain current and up-to-date information, as well as to gather and utilize the information they need [1]. In this study, we investigated the behaviors of clinical medical students regarding internet use and analyzed the characteristics of the students’ information demands. The results will be useful for improving library information services and for guiding and modifying internet use behaviors among clinical medical students.

## Methods

### Ethics statement

This study was approved by the Shengjing Hospital Ethics Committee (approval number of 2013PS168K). We obtained written informed consent for real-time feedback from all the participants.

### Source of participants

We recruited clinical medical students from an affiliated university teaching hospital to serve as the participants. The study sample included undergraduate medical students who were completing five years of study to obtain a bachelor’s degree in medicine for clinical practice (“five-year students”), undergraduate students who were completing seven years of study to obtain a master’s degree in medicine (“seven-year students”), master’s degree candidates who already had a bachelor’s degree in medicine (“master’s candidates”), and candidates for a doctoral degree in medicine (“doctoral candidates”).

### Design

We collected information for 31 continuous days. At regular times during this survey period, librarians requested users to provide a “Print Screen” as feedback of their online activities through an application program called gold disk electronic reading room system (GDERS). The data was recorded in a unified table. The feedback schedule was as follows: Monday through Thursday: 10:00 – 10:30, 12:30 – 13:00, 14:30 – 15:00, and 18:00 – 19:00; Friday: 10:00 – 10:30, 12:30 – 13:00, and 14:30 – 15:00; Saturday and Sunday: 10:00 – 10:30 and 14:30 – 15:00.

The table content was categorized into eight types of internet use: official work, obtaining literature resources, watching recreational videos, chatting and reading novels, online shopping, browsing a webpage, playing internet games, and other activities. For statistical analysis, the data were described as learning (official work and obtaining literature resources), leisure (watching recreational videos, chatting and reading novels, and browsing a webpage), e-commerce (online shopping), entertainment (playing internet games) and other activities in order to explain the online activities more briefly and coherently.

### Inclusion criteria and statistical methods

A total of 1484 all patrons responded and provided valid data during the 31-day survey period. In all, 999 of the enrollees were clinical medical students. The 999 related records were analyzed with the Excel program. The chi-square test was used to compare differences between groups. A p-value less than 0.05 was considered statistically significant.

## Results

### Student demographics and internet use behaviors

**Figure 1:**
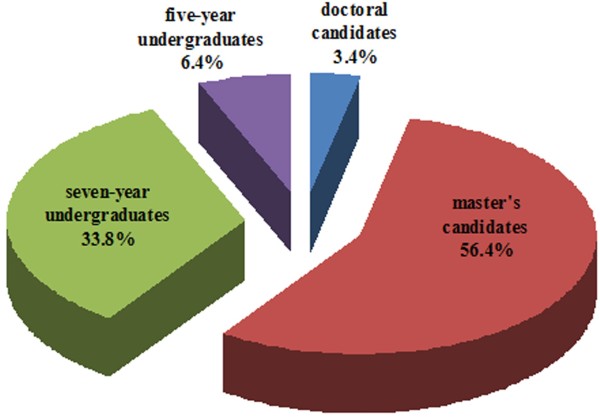
The proportion of clinical medical students in each academic level of study.

A total of 999 clinical medical students were enrolled in our study. In all, 34 (3.4%) of them were doctoral candidates, 563 (56.4%) were master’s candidates, 338 (33.8%) were seven-year undergraduates, and 64 (6.4%) were five-year undergraduates (Figure 1). Retrieving literature resources accounted for the highest proportion of internet use, but the behaviors varied among the different levels of clinical medical students. Table 1 presents the internet use behaviors of all students according to level of study.

**Table 1:** Frequency of eight internet activities according to academic level of clinical medical students

**Student category**	**Frequency (number of students) of internet activities**								
	**Official work**	**Obtaining literature resources**	**Watching recreational videos**	**Chatting and reading novels**	**Online shopping**	**Browsing a webpage**	**Internet-based games**	**Other**	**Total**
Doctoral candidates	5	20	2	1	0	5	1	0	34
Master’s candidates	94	174	40	51	37	123	35	9	563
Seven-year undergraduates	41	56	69	35	50	68	11	8	338
Five-year undergraduates	6	3	8	9	12	21	3	2	64
Total	146	253	119	96	99	217	50	19	999

### Distribution of internet use behaviors of different student groups

To facilitate statistical analysis, the pattern of internet use behaviors of clinical medical students was further categorized as learning, leisure, e-commerce, entertainment and other. The distribution of these activities according to level of study is provided in Table 2. There was a significant difference among the different levels of study of clinical medical students regarding use of the internet for learning (P < 0.0001). Doctoral candidates used the internet for learning most frequently (73.5%), followed by master’s candidates (47.6%). The proportions of seven- and five-year undergraduate students who used the internet for learning were 28.7% and 14.1%, respectively. A significant difference was also observed in the proportions of students who used the internet for leisure and e-commerce (P < 0.0001). The five-year undergraduate students used the internet for this purpose most frequently (59.4%), followed by seven-year undergraduate students (50.9%), master’s candidates (38.0%), and doctoral students (23.5%). Use of the internet for entertainment was similar among all clinical medical students (P > 0.05).

**Table 2:** Frequency of five categories of internet use according to academic level of clinical medical students

**Student category**	**Learning**		**Leisure**		**E-commerce**		**Entertainment**		**Other**	
	**No.**	**%**	**No.**	**%**	**No.**	**%**	**No.**	**%**	**No.**	**%**
Doctoral candidates	25	73.5	8	23.5	0	0.0	1	2.9	0	0.0
Master’s candidates	268	47.6	214	38.0	37	6.6	35	6.2	9	1.6
Seven-year undergraduates	97	28.7	172	50.9	50	14.8	11	3.3	8	2.4
Five-year undergraduates	9	14.1	38	59.4	12	18.8	3	4.7	2	3.1
Total	399	39.9	432	43.2	99	9.9	50	5.0	19	1.9

### The distribution of internet use during different time periods of the day

Table 3 lists the distribution of time of internet use among clinical medical students according to level of study. The daily period of internet use in the library was inconsistent among the groups of clinical medical students, but the difference was not significant (P > 0.05). Morning accounted for 18.3% of internet use, noon accounted for 36.2%, afternoon accounted for 30.2%, and night accounted for 15.2%. The noon and night use equaled 56.3% for seven-year students (38.8% and 17.5%, respectively), which was the highest proportion of use among any group of students. Doctoral students used the internet most often in the morning and afternoon (26.5% and 44.1%, respectively).

**Table 3:** Distribution of internet use during the day according to academic level of clinical medical students

**Student category**	**Morning**		**Noon**		**Afternoon**		**Night**	
	**No.**	**%**	**No.**	**%**	**No.**	**%**	**No.**	**%**
Doctoral candidates	9	26.5	10	29.4	15	44.1	0	0.0
Master’s candidates	101	17.9	201	35.7	176	31.3	85	15.1
Seven-year undergraduates	59	17.5	131	38.8	89	26.3	59	17.5
Five-year undergraduates	14	21.9	20	31.3	22	34.4	8	12.5
Total	183	18.3	362	36.2	302	30.2	152	15.2

### The distribution of internet use during different days of the week

Table 4 summarizes the distribution of day of internet use among clinical medical students according to level of study. The preferred day of internet use varied significantly among students (P < 0.01). Doctoral candidates used the internet most frequently on Tuesday and Wednesday (41.2% and 26.5%, respectively). Master’s candidates preferred to use the internet on Monday (19.7%).

**Table 4:** Distribution of internet use during the week according to academic level of clinical medical students

**Student category**	**Monday**		**Tuesday**		**Wednesday**		**Thursday**		**Friday**		**Saturday**		**Sunday**	
	**No.**	**%**	**No.**	**%**	**No.**	**%**	**No.**	**%**	**No.**	**%**	**No.**	**%**	**No.**	**%**
Doctoral candidates	5	14.7	14	41.2	9	26.5	2	5.9	4	11.8	0	0.0	0	0.0
Master’s candidates	111	19.7	104	18.5	89	15.8	78	13.9	63	11.2	69	12.3	49	8.7
Seven-year undergraduates	49	14.5	58	17.2	57	16.9	49	14.5	40	11.8	56	16.6	29	8.6
Five-year undergraduates	14	21.9	7	10.9	5	7.8	11	17.2	5	7.8	14	21.9	8	12.5
Total	179	17.9	183	18.3	160	16.0	140	14.0	112	11.2	139	13.9	86	8.6

### The daily and weekly distributions of internet use

**Figure 2:**
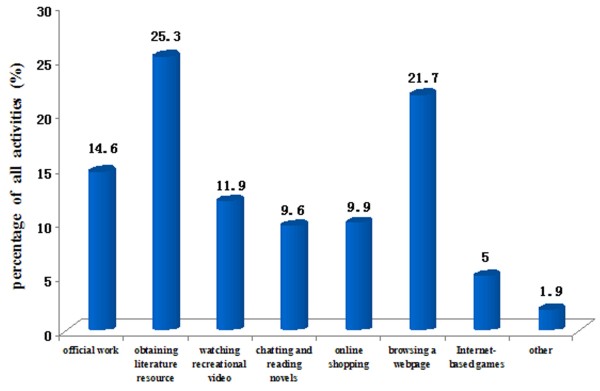
Distribution of internet use behaviors among all clinical medical students.

Figure 2 illustrates the percentages of all eight internet activities engaged in by clinical medical students. Searching for literature resources was the most common activity (25.3%), followed by browsing a webpage (21.7%) and official work (14.6%).

### The daily distribution of internet use

Table 5 shows the distribution of all online activities of clinical medical students according to time of day. There was no statistical difference among the time of day of internet use for learning, leisure and entertainment (P > 0.05). However, internet use for e-commerce was different throughout the day (P < 0.05). Learning was the primary activity (47.5%) in the morning, and leisure and e-commerce were most popular (48.0% and 13.2%, respectively) in the evening. Entertainment was most often reported at noon (5.5%). Overall, learning and leisure were the primary uses for the internet and together accounted for 83.1% of all use (39.9% and 43.2%, respectively).

**Table 5:** Distribution of internet activities in which clinical medical students engaged according to time of day

**Period**	**Learning**		**Leisure**		**E-commerce**		**Entertainment**		**Other**	
	**No.**	**%**	**No.**	**%**	**No.**	**%**	**No.**	**%**	**No.**	**%**
Morning	87	47.5	79	43.2	8	4.4	8	4.4	1	0.6
Noon	138	38.1	153	42.3	43	11.9	20	5.5	8	2.2
Afternoon	123	40.7	127	42.1	28	9.3	16	5.3	8	2.7
Night	51	33.6	73	48.0	20	13.2	6	4.0	2	1.3
Total	399	39.9	432	43.2	99	9.9	50	5.0	19	1.9

### The weekly distribution of internet use

Table 6 provides the distribution of all online activities of clinical medical students according to day of the week. The use of the internet for learning and e-commerce did not differ by day of the week (P > 0.05), but the distributions of leisure and entertainment activities did vary by day (P < 0.05). Internet use for learning was highest on Friday (43.8%) and Sunday (43.0%) and leisure activities were highest on Tuesday (49.7%) and Saturday (51.1%). Moreover, e-commerce and entertainment use represented 13.4% and 10.1%, respectively, of all use on Monday, which represented the highest proportions of these activities on any day of the week.

**Table 6:** Distribution of internet activities in which clinical medical students engaged according to day of the week

**Day**	**Learning**		**Leisure**		**E-commerce**		**Entertainment**		**Other**	
	**No.**	**%**	**No.**	**%**	**No.**	**%**	**No.**	**%**	**No.**	**%**
Monday	69	38.6	65	36.3	24	13.4	18	10.1	3	1.7
Tuesday	66	36.1	91	49.7	19	10.4	6	3.3	1	0.6
Wednesday	67	41.9	73	45.6	15	9.4	5	3.1	0	0.0
Thursday	59	42.1	53	37.9	15	10.7	10	7.1	3	2.1
Friday	49	43.8	41	36.6	11	9.8	4	3.6	7	6.3
Saturday	52	37.4	71	51.1	10	7.2	3	2.2	3	2.2
Sunday	37	43.0	38	44.2	5	5.8	4	4.7	2	2.3
Total	399	39.9	432	43.2	99	9.9	50	5.0	19	1.9

## Discussion

For this study, librarians collected data to record clinical medical students’ online activities. The recorded data was statistically analyzed, which provided an overview of internet use behaviors of clinical medical students. This research intended to explore whether there were any differences among the choice of internet-based activities of clinical medical students. We also examined whether time of day or day of the week influenced the use of internet activities.

### Internet use among clinical medical students

Graduate students composed the largest segment of the survey respondents. Clinical medical students’ needs for learning varied according to their level of education. A stronger need for learning was associated with a higher academic level of study. Learning-related activities accounted for an average proportion of 39.9% among all respondents. This figure is lower than data previously reported by Geng and Zhu [2,3]. Specifically, Geng’s study showed that accessing document information represented 64.7% of internet use in an electronic reading room. Clinical medical students’ choices of activities varied among the five broad categories of internet use. For example, master’s candidates and doctoral candidates preferred learning-related activities when they were online in the library. All of the respondents had a demand for entertainment and the use of the internet for entertainment was consistent across all student groups. We also observed that five-year students did not have a strong need for information and this group had a lower frequency of information-related activities. These results are similar to reports from a previous study by Yu in 2009 [4]. The findings of the present study suggest that the use of the internet by clinical medical students for learning-related activities varied according to the different educational backgrounds, which agrees with previous research that reported that the use of electronic resources differs among students [5]. Therefore, a personalized information resource service should be offered to meet the needs of different user groups within a library. This concept is consistent with the idea of reader-centered services. Moreover, libraries should consider users’ academic levels when building the internet-based information resources of the library [6].

### Time of internet use among clinical medical students

Although the results showed no statistical differences in the time of day of internet use for the entire sample of clinical medical students, each group showed different patterns of online use according to time of day. Each group of clinical medical students chose their times in the library according to personal schedules. For example, doctoral candidates most frequently preferred to use the library during working hours, while personal spare time during non-working hours was most popular with seven-year students. These schedule differences may be related to the current training pattern that offers priority to scientific research in doctoral graduate education, while clinical practice is the primary focus for seven-year students. Differences in internet use were observed among the student groups according to day of the week. Specifically, doctoral candidates used the internet most often on Tuesday and Wednesday and five-year students used the internet most often on the weekend, which is likely due to course arrangement and schedule. The seven-year students and master’s candidates showed no significant differences in the distributions of internet use according to day of the week.

### Internet use according to time of day and day of week

Internet-use behavior was different according to time of day and day of the week. Internet use for learning, leisure and entertainment did not differ during the times of the day, but e-commerce activity differed significantly among the different times of day. The time of day for internet use did vary among all types of online activities. Learning occurred most often in the morning, while leisure and e-commerce activities were popular in the evening. Noon was the most popular time for entertainment activities. Learning and leisure activities accounted for 83.1% of all internet activities (39.4% and 43.2%, respectively).

Learning and e-commerce activities did not vary according to day of the week, but leisure and entertainment activities varied significantly among the days of the week. Leisure activities accounted for 49.7% and 51.1% of internet use on Tuesday and Saturday, respectively. Entertainment time accounted for 10.1% of internet use on Monday.

Internet-use behaviors differed according to times of day and day of the week. The distribution of learning activities was consistent in a single day and across the days of the week. The distribution of leisure activities was consistent throughout the times of the day but varied by day of the week. Similarly, entertainment activities were evenly distributed during a single day but differed among the days of the week. The distribution of e-commerce activities varied within a single day but did not differ among the days of the week.

## Conclusions

In conclusion, librarians should provide guidance for internet use to clinical medical students, including offering reasonable schedules, displaying positive attitudes, obtaining learning information technology, applying information knowledge thoughtfully, and encouraging students’ abilities for independent learning. Reasonable rules and regulations should also be prepared that contribute to improved learning efficiency and enhanced use of internet-based resources among clinical medical students.

## Competing interests

The authors declare that they have no competing interests.

## Authors’ contributions

YH designed the research and drafted the manuscript. Z LQ participated in the design of the study and statistical analysis. C YX, XX, CX collected the student feedback and completed the data collection. All the authors read and approved the final manuscript.

## Pre-publication history

The pre-publication history for this paper can be accessed here:

http://www.biomedcentral.com/1472-6920/14/67/prepub
